# How does psychological resilience influence subjective career success of Internet marketers in china? A moderated mediation model

**DOI:** 10.3389/fpsyg.2022.921721

**Published:** 2022-08-01

**Authors:** Ting Wang, Di Gao

**Affiliations:** Business School, China University of Political Science and Law, Beijing, China

**Keywords:** psychological resilience, Internet marketers, subjective career success, work engagement, workload

## Abstract

In this study, the research objective of psychological resilience refers to the emerging professional group of Internet marketers under the background of the COVID-19 pandemic environment. This paper studies the effect of the psychological resilience of Internet marketers on their subjective career success. The result shows that Internet marketers’ psychological resilience has a positive impact on their subjective career success. The work engagement of Internet marketers plays a mediating role in the relationship between psychological resilience and subjective career success. Meanwhile, Internet marketers’ workload positively moderates the mediating effects of work engagement. This study starts from the perspective of positive psychology to investigate the psychological resilience of Internet marketers and broadens the scope of application of positive organizational behavior and psychology.

## Introduction

Since the 1970s, the internal and external working environments of organizations have changed rapidly, and the boundaries of their survival and development have increasingly blurred (Fu and Chen, 2022). Hence, employees cannot clearly determine the underlying values, psychological contracts, hypothetical systems, and the specific content of rights and responsibilities that guide their work. Individuals find highly competitive external work environments extremely stressful or unhealthy. Then, against the backdrop of the current flattened world economy, there is a need for research on the psychological resilience of employees. Moreover, China is in a critical period of transformation of its economic development mode at present, new employment modes and labor patterns are constantly emerging (Yan and Yang, 2022). Internet marketers, as a typical representative of the new form of employment, are the main drivers of stable employment in China during the ongoing COVID-19 pandemic.

In the combination of live broadcast and e-commerce industry, online marketers have experienced the appellation from Internet celebrities to live broadcast anchors. In July 2020, the Ministry of Human Resources and Social Security of China and other relative departments listed Internet marketers as a new occupation. Meanwhile, the professional title of Internet marketer was officially included in the “National Occupational Classification Code” (Wu, 2021). Based on the interactive nature of the Internet and the characteristics of the credibility of transmission, an Internet marketer refers to a person who sells and promotes products on a digital platform (Shen, 2020). It has been nearly 6 years since the outbreak of live broadcasting. As an emerging profession, Internet marketers have developed tremendously along with the continuous improvement and perfection of Internet technology. Obviously, the continuity of its professional life cycle has stood the test of time, and Internet marketers are increasingly recognized by the society.

Additionally, with the vocational certification of the Ministry of Human Resources of China and Social Security and the State Administration of Market Regulation in China, Internet marketers are being increasingly recognized by the masses. However, the novelty of the work content of Internet marketers, coupled with the fierce changes in the internal and external environment, makes the Internet marketing profession frequently bear various hidden burdens, and psychological–emotional exhaustion often has a negative impact on their personal and work development. The exploration and improvement of the quality of psychological resilience help individuals reflect on the significance and value of life brought by adversity.

### Literature review

#### Psychological resilience

Psychological resilience is a static situation (Masten and Obradovic, 2006) wherein an individual can maintain a benign development after setbacks and negative emotions or events, or it is the ability to recover through one’s own initiative under violent and destructive external changes to overcome adversity and rebound from setbacks (Mak et al., 2011; Zhou et al., 2016). Empirical research on resilience found that whether in the field of clinical psychology or that of positive psychology, resilience plays an important role in the adaptation, recovery, and physical health of individuals after trauma (Lee et al., 2013). Studies have highlighted that an individual with high psychological resilience, as a typical representative of psychological capital ability, can overcome internal and external pressure, cooperate with others, and assume the corresponding risks as appropriate (Han et al., 2021).

#### Work engagement

Work Engagement (WE) refers to the positive emotional experience that an individual perceives in daily work, which is characterized by long durations and multidimensional divergence to the individual’s work process (Lin et al., 2008). According to the work requirement–resource model, the factors that affect the degree of work engagement generally include employees’ own resources and resources needed for work, among which individual resources refer to self-efficacy, resilience and optimism, and other individual positive trait variables. Psychological capital theory also proposes that an individual’s psychological capital resources can stimulate them to generate positive achievement motivation and strengthen work identity to ultimately increase the degree of work engagement (Cheung et al., 2011; Clark et al., 2021). A high level of work engagement means that an individual will have more positive emotions and feelings toward work completion (Bakker et al., 2008; Awan et al., 2020). Similarly, an individual’s positive mental state can also be reflected in the elements of psychological capital. Employees with high psychological capital are more optimistic and positive in their work attitude and will create more value for the society. Specifically, the work engagement-related effect variables mainly include job satisfaction (Guo et al., 2016), job performance (Li et al., 2015), turnover tendency (Yang et al., 2017), and organizational citizenship behavior (Diefendorff et al., 2002; Geng and Wei, 2016).

#### Workload

Workload refers to the index value of an individual’s ability to evaluate workload demand (Wang and Li, 2017) or the sum of the full cost of completing work requirements, which is the integration of subjective cognition and objective resources required by individuals to perform tasks (Veltman and Gaillard, 1996; Geng et al., 2020). In the existing domestic and foreign literature, although some scholars have proposed workload as an important “challenging” stressor, it can play a role in motivating individuals to work hard (Cavanaugh et al., 2000). Empirical studies have shown that workload has a negative impact on the level of an individual’s cognition and organizational growth (Sonnentag and Fritz, 2014). From the perspective of an individual’s work attitude and wellbeing, the impact of workload on work performance, job satisfaction (Nirel and Feigenberg, 2008; Häusser et al., 2010), and work engagement all have negative effects (Weigl et al., 2016). From the perspective of organizational profit and growth, workloads clearly positively affect an employees’ turnover tendency (Torres, 2016). As a typical occupation in the live broadcast industry represented by emerging industries, Internet marketers often have to undertake a relatively large workload. An empirical study on work requirements and engagement found that tasks that arouse individual motivation have a significant positive effect on work engagement, whereas depressive tasks have a significant negative effect (Zhang and Wang, 2017). Furthermore, specifically, challenging stressors—such as high workload, time pressure, scope of work, and responsibilities—also bring material and spiritual expectations to employees when they consume numerous personal resources, such as wage increases, personal growth, and self-development (Crawford et al., 2010).

### Theoretical foundation

#### Psychological capital theory

The formation and value recognition of psychological capital concept benefit from the deepening of research on organizational behavior. Psychological capital pays more attention to the development of “people” as a whole, and focuses on the characteristics and development of individuals (Luthans et al., 2005). Like traditional human capital and social capital, psychological capital also covers group-level content such as talent, professional skills and practical experience, as well as social support and network. However, psychological capital focused on the process of the individual’s transformation from the actual self to the potential self-more. During a career development without boundaries, the overall effect of human, social and psychological capital is the key to realize the potential power of people. Compared with single capital, the synergistic effect of three different types of capital will have a more significant impact. Whether at the individual level or at the overall one, psychological capital theory helps modern organizations to understand modern human resources and their engagement more comprehensively. As an important research basis of organizational behavior, psychological science also affects the research orientation of organizational behavior to a certain extent. Positive psychology advocates people to focus on positive power or potential. It believes that individuals need to properly treat psychological problems, in order to maintain a normal mental health state.

#### Resource conservation theory

The resource conservation theory proposed by Hobfoll (1989) is mainly used to explain the back-and-forth process of resources between individuals and their environment. The most basic assumption of resource conservation theory is that when people face actual or potential resource loss, they will preserve, maintain and acquire precious resources as much as possible in order to eliminate the threat of resource loss. There are three important interrelated corollaries of resource conservation theory: resource conservation priority, resource acquisition secondary, and creation of additional resources. Resource protection priority means that when resources are already lost, the individual awareness of protecting the resources he owns is higher than acquiring additional resources. Resource acquisition secondary means that the motivation of individuals to acquire resources is weaker than the motivation to save resources, but it does not mean that acquiring additional resources is not desirable. On the one hand, the acquisition of resources can reduce the risk of actual or potential loss. On the other hand, it can create opportunities for acquiring additional cherished resources. Finally, creating additional resources means that individuals will use multiple ways to strive for additional cherished resources in order to gain more resources. As a source of stress to consume resources, work stress will stimulate the motivation of individuals to protect resources. However, the latest research points out that the motivation of individuals to acquire resources can also be stimulated when resources are exhausted. Therefore, work stress will stimulate resource conservation or resource acquisition motivation, depending on which motivation is more conducive to coping with resource depletion.

### Hypothesis development

#### Psychological resilience and subjective career success

When individuals have high psychological resilience, they can show higher engagement at work, are more likely to develop good interpersonal relationships, improve their work remuneration and promotion opportunities, and feel additional work satisfaction. It has confirmed that psychological resilience and job performance show a positive correlation, be it in normal or extreme working environments, such as military combat (Schaubroeck et al., 2011). In conclusion, resilient individuals can solve problems faster and better, overcome obstacles, and eventually achieve considerable career development. Thus, in our hypothesis, we consider Internet marketers as the research object to study the influence of psychological resilience on their subjective career success. Specifically, we propose the hypothesis 1:

**Hypothesis 1**: Internet marketers’ psychological resilience has a positive impact on their subjective career success.

#### Mediating role of work engagement

Nowadays, although the webcast industry is increasingly becoming standardized, there are few ways to pass positive information to the public; some people retain the stereotype of “vulgarization” and “stigmatization” of the group, thus leading to a low degree of social identity for a career as an Internet marketer. For a group of Internet marketers, the relative lack of government-level social security systems and the negative propaganda of public opinion in the media have caused Internet marketers to suffer serious frustrations in the course of their work. In the face of injury or dilemma, individuals with strong resistance to frustration continue to maintain a high degree of daily work engagement, whereas groups with low levels of relative psychological resilience are unable to concentrate on work because of the influence of negative emotions. Empirical research on psychological capital recognizes that psychological capital must be related to an organization’s work results. However, work attitude variables, such as work engagement and satisfaction, also have a positive correlation with job performance outcome variables. Thus, psychological capital and employees’ work engagement are related. Individuals with a high level of psychological resilience can maintain a higher degree of engagement in their own work, which is reflected in the focus, higher work willingness, and high cost in the professional field. Specifically, we propose the hypothesis 2:

**Hypothesis 2:** Internet marketers’ work engagement plays a mediating role in the relationship between psychological resilience and subjective career success.

#### Moderating role of workload

Scholars have debated about the role of workload in the transfer mechanism of empirical research. According to Hockey’s work demand–management control model, when there is psychological and emotional stress in the working environment, employees choose to enhance their subjective initiative, that is, to continue working hard to maintain high performance levels. Thus, the higher the degree of activation of sympathetic nerves in the brain when an individual chooses to strengthen subjective effort, the higher the psychological cost it produces. Hence, in the long term, when individuals face a highly intense working environment, one is more likely to experience situations where one’s own state makes it difficult to meet the needs of work, resulting in job burnout. Scholars have focused on the work demand–management model and believed that individuals appear to deal with highly demanding work requirements in two ways, namely, one is paying a higher psychological cost to adapt to the high requirements and complete the higher target performance and the other is avoiding excessive consumption to maintain normal effort input. However, workload reduces the final level of work performance. Specifically, we propose the hypothesis 3:

**Hypothesis 3:** Internet marketers’ workload positively moderates the mediating effects of work engagement in the relationship between psychological resilience and subjective career success.

**FIGURE 1:**
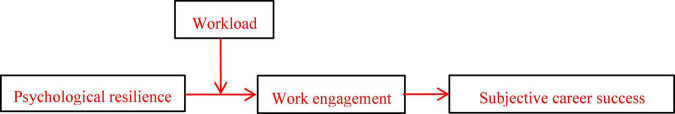
Theoretical research model.

To sum up, the theoretical model and research hypotheses of the relationship between psychological resilience, work engagement, workload and subjective career success constructed in this paper are shown in Figure 1 and Table 1.

**TABLE 1:** Research hypotheses summary.

Hypotheses	Hypothetical content
Hypothesis1	Internet marketers’ psychological resilience has a positive impact on their subjective career success.
Hypothesis2	Internet marketers’ work engagement plays a mediating role in the relationship between psychological resilience and subjective career success.
Hypothesis3	Internet marketers’ workload positively moderates the mediating effects of work engagement in the relationship between psychological resilience and subjective career success.

## Materials and methods

### Research samples

In this study, the selection of the survey object is not limited to a fixed platform. The source of the data is mainly live broadcast bases in Lianyungang, Jiangsu, and Shiyan, Hubei, as well as the Internet marketers from many provinces such as Beijing, Shanghai, and Shenzhen. Additionally, an online questionnaire was adopted as the survey method. The questionnaire was distributed and collected primarily through the WeChat platform. Overall, 240 questionnaires were distributed, of which 224 responses were collected. The questionnaire response rate was 93.3%. After inspection, 16 invalid questionnaires were excluded and 208 valid questionnaires were retained. The effective recovery rate reached 92.8%. All research subjects completed the survey independently.

### Variable measurements

In this study, the concept of psychological resilience is defined as a trait or ability. The Connor–Davidson Resilience Scale (CD-RISC) (Connor and Davidson, 2003) is used to measure the psychological resilience of Internet marketers in this paper. This scale is used to assess the positive psychological traits that individuals display in difficult situations to overcome adversity and eventually achieve personal growth. The CD-RISC is currently the most commonly adopted scale to assess resilience levels. However, because the design background of CD-RISC is based on European and American cultures, Chinese scholars developed psychological resilience measurement tools suitable for local research on the basis of Chinese culture (Yu and Zhang, 2007). CD-RISC divides psychological resilience into three dimensions, namely, tenacity (I can achieve my goals), self-improvement (I can adapt to changes), and optimism (I can cope with whatever happens), with 25 items. Using the Richter seven-point scale, the scoring method ranges from 1, “strongly disagree,” to 7, “strongly agree.” That is, the higher the score of the scale, the stronger the resilience of the individual. Cronbach’s coefficient of the Chinese version of the resilience scale reached 0.91. The three-factor structure is properly divided, which means that the adopted scale shows good validity of the method for analysis.

The measurement scale used in this study to assess the level of work engagement is the Utrecht Work Engagement Scale (UWES-9), a simplified version of UWES (González-Romá et al., 2006). The scale is compiled and revised on the basis of the work engagement model, which specifically includes three factors: vitality, dedication, and focus (Wilmar et al., 2002). The items on the vitality dimension are used to assess an individual’s strength, level of flexibility, willingness to devote time, as well as the inability to easily burnout, and firmness and maintenance in the face of difficulties, such as “At work, I perceive myself bursting with energy.” The items on the dedication dimension are used to evaluate the value that an individual obtains from one’s career, passion and pride towards the work, such as “I am proud of my own career.” The items on the focus dimension are used to assess the level of the test subject’s full immersion in their work, such as “When the work is stressful, I feel happy.” In a study on specific objects, Bakker et al. (2008) showed that the amount of work engagement has good reliability and validity. Additionally, the Chinese version of the Work Engagement Evaluation Scale (Zhang and Gan, 2005) scores is relatively stable. Among them, vitality, dedication, and focus have good internal consistency, and all Cronbach’s coefficients exceed 0.70.

Role Overload Scale (Peterson et al., 1995) is used in this study to assess the level of workload. Peterson developed the workload sense assessment scale on the basis of the research content in their cross-country literature study on role conflict, role ambiguity, role overload. There are five items in the workload scale, such as “my work quantity hinders the quality of work I want to maintain.” In the subjective perception of workload, Internet marketers have no significant professional characteristics; hence, all scale items in this study are consistent with the Chinese-translated items of the original scale. The Cronbach’s coefficient of the role overload scale is 0.88.

Subjective career success is an individual’s subjective perception of the success of their own career development. An individual should make a subjective measurement on the basis of their own practical experience and feelings depending on specific measurements. It is difficult to obtain the result through the organization engaged in or relying on external observation of a third party. Based on the conceptual connotation of subjective career success, many scholars have used career satisfaction (Greenhaus et al., 1990) or job satisfaction to measure a subject’s perceived subjective career success. The Subjective Career Success Measurement Scale used in this study has a five-item scale developed by Greenhaus (such as “I have satisfaction with the success of my career”) that fits with the definition of subjective career success, which measures the level of individual subjective career success mainly by career satisfaction indicators. The reliability and validity of the subjective career success scale of Greenhaus, which is also a widely used subjective career success scale, were robust and were supported and verified by several sample data in China. The Cronbach’s coefficient of the subjective occupational success scale reached 0.95.

### Data analysis

In this study, SPSS25.0 and AMOS 24.0 software were used to analyze the data. Pearson’s correlation analysis method was used to observe the correlation between sex, educational background, and other statistical variables such as psychological resilience, work engagement, workload, and subjective career success. To test the mediation and moderation effects, we adopted a bootstrapping method, used the program in SPSS developed by Preacher and Hayes (2008), and selected Model 4 in the PROCESS3.0 plug-in to test the mediation effect and Model 7 in the PROCESS3.0 plug-in to examine the moderating effect. Specifically, we set a random sampling at 5,000 times and used a 95% confidence interval for testing, to obtain the specific values of the mediation and moderation effects.

## Results

### Descriptive statistics

The questionnaires for this study are mainly from two live broadcast bases in Lianyungang, Jiangsu, and Shiyan, Hubei. We distributed questionnaires to 208 Internet marketers from Beijing, Shanghai, Shenzhen, Anhui, and other provinces. Descriptive statistical analysis is performed on the basis of the data, and the details are shown in Table 2.

**TABLE 2:** Demographic information of valid survey sample composition (*N* = 208).

Control variable	Sample characteristic content	Frequency number	Effective percentage	Accumulated percentage
Sex	Male	79	37.90	37.90
	Female	129	62.10	100
Age	Under 20 years	0	0	0
	21–25 years	52	25	25
	26–30 years	41	19.71	44.71
	31–35 years	47	22.60	67.31
	36–45 years	41	19.71	87.02
	Over 46 years	27	12.98	100
Education	High school and below	0	0	0
	High school	10	4.81	4.81
	College	154	74.04	78.85
	College undergraduate	42	20.19	99.04
	Master’s degree	2	0.96	100
	Doctor’s degree	0	0	100
Working years	Less than a month	36	17.31	17.31
	1–6 months	45	21.63	38.94
	6 months–1 year	38	18.27	57.21
	1–3 years	59	28.37	85.58
	3–5 years	8	3.85	89.43
	Over 5 years	22	10.57	100
Income	Under RMB 2,000	9	4.33	4.33
	RMB 2,000–3,000	12	5.77	10.10
	RMB 3,000–4,500	34	16.35	26.45
	RMB 4,500–6,000	34	16.35	42.80
	RMB 6,000–8,000	24	11.54	54.34
	RMB 8,000–10,000	34	16.35	70.69
	RMB 10,000–15,000	28	13.46	84.15
	RMB 15,000–20,000	21	10.10	94.25

In terms of sex, 79 were men and 129 women, accounting for 37.9% and 62.1% of the total sample respectively. The data results show that the proportion of men and women in the survey questionnaire is significantly biased—that is, there are significantly more female Internet marketers than males, which is consistent with the phenomenon observed in real society. In terms of age, none were under the age of 20; 52 aged 21–25 years, accounting for 25% of the total sample; 41 aged 26–30 and 36–45 years, accounting for 19.71% of the total sample; 47 under the age of 31–35 years, accounting for 22.6% of the total sample; and 27 over 46 years, accounting for 12.98% of the total sample. Therefore, in terms of the proportion of age, the age of Internet marketers is at a maximum of 21–25 years, indicating that more youngsters are engaged in this profession, which is consistent with the characteristics of this profession as a representative of flexible employment. In terms of academic qualifications, the number of Internet marketers with college as the highest academic qualification is the highest (154), accounting for 74.04%, followed by undergraduate and high school levels, accounting for 20.19% and 4.81% of the total sample, respectively. There are none with a degree below the high school level or with a doctor’s degree, indicating that the education background of the group currently engaged in this occupation represents a normal distribution, and the number of people with higher and lower education levels is still small. In terms of working hours, the number of people with entry time between 1 and 3 years is the highest, accounting for 28.37% of the total sample proportion, followed by those with entry time between 1 and 6 months and from 6 months to 1 year, with 45 and 38 people, accounting for 21.63% and 18.27% of the total sample, respectively. This indirectly proves that the life cycle of Internet marketers as a new profession has withstood the test of time. In terms of income, 89 people with a salary of less than 6,000 yuan account for 42.80% of the total sample; 58 people with a salary between 6,000 and 10,000 yuan account for 27.89% of the total sample; and 61 people with a salary between 10,000 and 30,000 yuan account for the total sample. The sample proportion is 29.33%. Hence, the income of most groups of Internet marketers remains low, which is consistent with the phenomenon that the top current Internet marketers are still earning maximum resources and traffic in the industry. It also reflects the grassroots Internet. Hence, marketers find it difficult to obtain traffic that attracts the masses.

### Correlation analysis between variables

Pearson’s correlation analysis between variables is used in this study to observe the correlation between sex, education, and other statistical variables such as psychological resilience, work engagement, workload, and subjective career success. Table 3 lists the mean (mean), standard deviation (standard error), and correlation coefficient (correlation index) between the control and main research variables. The control variables include the sex, age, education, working years, and income. As shown in Table 3, psychological resilience and work engagement (*r* = 0.444, *p* < 0.01), psychological resilience and subjective career success (*r* = 0.441, *p* < 0.01), work engagement and subjective career success (*r* = 0.496, *p* < 0.01), work engagement and workload (*r* = –0.225, *p* < 0.01), and workload and subjective career success (*r* = –0.189, *p* < 0.01) are all significantly correlated. The correlation coefficient values of the main research variables in Table 3 show that the correlation between each variable is consistent with the prediction hypothesis, and it also provides a preliminary stage of support for the following hypothesis.

**TABLE 3:** Mean, standard deviation, and correlation coefficients of the study variables.

Variable	Mean	Standard deviation	1	2	3	4	5	6	7	8
(1) Sex	0.524	0.501	1							
(2) Age	2.760	1.366	–0.098	1						
(3) Education	2.995	0.577	–0.008	–0.069	1					
(4) Working years	2.115	1.515	–0.067	–0.017	0.078	1				
(5) Income	4.130	2.137	0.035	0.087	0.345**	0.092	1			
(6) Psychological resilience	4.723	1.579	0.083	0.025	0.334**	–0.004	0.246**	1		
(7) Work Engagement	5.277	1.542	–0.006	0.040	0.653**	0.070	0.566**	0.444**	1	
(8) Subjective career success	5.330	1.513	0.011	0.117	0.402**	0.005	0.252**	0.441**	0.496**	1
(9) Workload	4.301	1.884	–0.003	0.059	–0.231**	–0.064	–0.181**	–0.156*	–0.225**	–0.189**

N = 208; ** represents p < 0.01.

### Hypothesis test

In the process of formal research, linear regression method is used to verify assumptions. The results are shown in Table 4. After controlling for variables such as sex, age, education, working years and income of Internet marketers, regression analysis of psychological resilience to subjective career success is performed. We found a significant positive correlation between psychological resilience of Internet marketers and subjective career success (β = 0.310, *p* < 0.001) after controlling for variables such as sex, age, education, working years, and income. Hypothesis 1 is supported—that is, the psychological resilience of Internet marketers has a positive impact on subjective career success. Additionally, before testing the mediation effect of work engagement, it is necessary to analyze the relationship between the psychological resilience and work engagement, as well as the relationship between work engagement and subjective career success. The results showed that after controlling for the variables of sex, age, education, working years, and income of Internet marketers, a significant positive correlation was found between the psychological resilience of Internet marketers and work engagement (β = 0.186, *p* < 0.001),and between work engagement and subjective career success of Internet marketers (β = 0.291, *p* < 0.001).

**TABLE 4:** Results of regression analysis for hypothesis testing.

	Work engagement		Subjective career success			
	Model 1		Model 2		Model 3	
	B	*S.E.*	β	*S.E.*	β	*S.E.*
Sex	–0.028	0.138	–0.006	0.181	0.002	0.178
Age	0.026	0.051	0.118	0.067	0.111	0.066
Education	0.447***	0.135	0.256***	0.176	0.126	0.204
Working years	–0.007	0.046	–0.029	0.060	–0.027	0.059
Income	0.323***	0.036	0.036	0.047	–0.058	0.051
Psychological resilience	0.186***	0.047	0.310***	0.062	0.256**	0.063
Work engagement					0.291***	0.091
Workload	–0.036	0.038	–0.084	0.050	–0.073	0.049
Psychological resilience × workload	0.126**	0.077	0.103	0.101	0.066	0.101
R square	0.609		0.302		0.336	

N = 208, ** represents p < 0.01; *** represents p < 0.001.

To test the mediating role of work engagement in Hypothesis 2, we apply bootstrapping methods using the method proposed by Preacher and Hayes (2008). Specifically, we set random sampling at 5,000 times using a 95% confidence interval. As shown in Table 5, the results of regression analysis show that when psychological resilience is an independent variable, the prediction of work engagement proves that the test results of the first half of the mediation effect are true (β = 0.198, *p* < 0.001). Second, from the results of psychological resilience and work engagement to predict subjective career success, psychological resilience significantly positively predicts subjective career success (β = 0.254, *p* < 0.001). Meanwhile, Table 5 shows that work engagement significantly positively predicts subjective career success (β = 0.307, *p* < 0.001), indicating that the direct effect and the second half of the mediation effect are established. Finally, the subjective career success of dependent variables is predicted using psychological resilience as an independent variable. Table 5 shows that the β = 0.315, *p* < 0.001, indicating that the total effect is true.

**TABLE 5:** Mediation test of work engagement.

	Work engagement		Subjective career success		Subjective career success	
	β	*P*	β	*p*	β	*p*
Sex	–0.0856	0.5415	0.0073	0.9670	–0.0190	0.9172
Age	0.0380	0.4612	0.1230	0.0613	0.1347	0.0458
Education	1.2423	0	0.3510	0.0853	0.7329	0
Working years	0.0008	0.9868	–0.0205	0.7260	–0.0203	0.7357
Income	0.2549	0	–0.0315	0.5310	0.0469	0.3081
Psychological resilience	0.1980	0	0.2538	0.0001	0.3146	0
Work engagement			0.3074	0.0007		
R square	0.595		0.328		0.288	
F	49.191		13.921		13.541	

To further detect the mediation effect as a complete or partial mediation, and whether it is a partial mediating role, the specific value of the mediation effect is obtained. The random sampling is set to 5,000 times using bootstrap random sampling, and the confidence interval is 95%. Finally, as shown in Table 6, the total effect is 0.315, the confidence interval is [0.193, 0.436], which means the total effect is significant. The direct effect is 0.254, the confidence interval is [0.131, 0.377]. The mediating effect is 0.061 and the confidence interval is [0.023,0.125], indicating that the mediating effect is true. Moreover, as shown in Table 6, the proportion of mediating effect to total effect is 19.36%. That is, the work engagement of Internet marketers plays a 19.36% mediating role between psychological resilience and subjective career success.

**TABLE 6:** Decomposition of the total, direct, and mediating effects.

	Effect	Boot SE	LLCL	ULCL	Percentage of effects (%)
Total effects	0.315	0.062	0.193	0.436	100
Direct effects	0.254	0.063	0.131	0.377	80.64
Mediating effects	0.061	0.025	0.023	0.125	19.36

**FIGURE 2:**
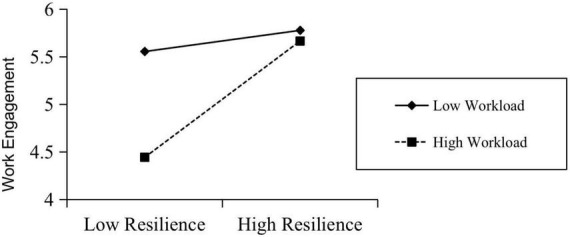
Moderation effect of workload.

The method proposed by Hayes (2013) is used to test this moderation effect. Specifically, we set random sampling to 5,000 times using a 95% confidence interval. Consequently, the specific value of the moderated mediating effect is obtained, the results of which are shown in Table 7. The data of the model as a whole showed a moderated effect index of moderated mediation at 0.056 and 95% CI index of moderated mediation [0.114, 0.098]. As the confidence interval in which the value is located does not contain 0, the moderation index in the model is said to be significant. Hence, this study proves that the mediation effect in the model constructed can be effectively moderated. Specifically, the amount of mediation effect in the sample of –1 SD is 0.002, with 95% CI [–0.087, 0.099]. The results showed that the mediation effect was not established in the moderated low-group sample, and the mediation effect was 0.236,with 95% CI [0.114, 0.098] in the +1 SD sample, which indicates that the mediation effect holds good for the high grouping of moderated variables. And the corresponding moderation effect diagram is shown in Figure 2. Besides, all the research hypotheses and test results are shown in Table 8.

**TABLE 7:** Results of conditional indirect effects.

Dependent variable	Adjustment variable	Effect	Bootstrapped SE	Bootstrapped 95% CI
Subjective career success	–1 SD	0.002	0.047	[–0.087, 0.099]
	Mean	0.180	0.051	[0.088, 0.287]
	+ 1 SD	0.236	0.066	[0.114, 0.375]
	Index of moderated mediation	0.056	0.019	[0.114, 0.098]

**TABLE 8:** Research hypotheses and test results.

Hypotheses	Hypothetical content	Test results
Hypothesis 1	Internet marketers’ psychological resilience has a positive impact on their subjective career success.	Valid
Hypothesis 2	Internet marketers’ work engagement plays a mediating role in the relationship between psychological resilience and subjective career success.	Valid
Hypothesis 3	Internet marketers’ workload positively moderates the mediating effects of work engagement in the relationship between psychological resilience and subjective career success.	Valid

## Conclusion

On the basis of a literature review, this study proposes three related hypotheses and validates them through data analysis using statistical analysis software. The results show that the psychological resilience of Internet marketers has a significant positive impact on subjective career success; the work engagement of Internet marketers plays a mediating role between psychological resilience and subjective career success; and the workload of Internet marketers positively moderates the relationship between psychological resilience and subjective career success. Specifically, it is manifested in the following three aspects.

First, the psychological resilience of Internet marketers improves their subjective level of career success cognition. This study uses psychological capital theory as the theoretical basis and highlights that individuals with high psychological resilience can overcome internal and external pressures and have a higher sense of self-efficacy and innovative spirit. This enables them to better solve problems, cope with adversity, and actively adapt to the results, which ultimately bring success, satisfaction, and development to the individual. Additionally, the results of the data analysis by SPSS 25.0 statistical analysis software show that the Internet marketers’ psychological resilience has a significant positive impact on subjective career success (β = 0.310, *p* < 0.001). Psychological resilience is a typical positive psychological resource. Under the effect of this positive psychological trait, individuals show stronger adaptability in the work process, are more likely to develop good interpersonal relationships, and be satisfied with work. The perceived happiness also increases accordingly, which means that it is easier for Internet marketers to achieve subjective career success.

Second, the work engagement of Internet marketers plays a mediating role between psychological resilience and subjective career success. On the basis of literature review, this study confirms that a moderation effect exists between Internet marketers’ psychological resilience and subjective career success in relation to work engagement. The empirical research results show that work engagement has a partial mediating effect between the psychological resilience of Internet marketers and subjective career success (β = 0.291, p < 0.001). Work engagement is derived from the perspective of positive psychology and organizational behavior, because it reflects the positive and full emotions and cognitive patterns of an individual at work and indicates that the individual can perceive the enthusiasm and joy of work when completing the task—that is, it has a higher sense of self-fulfillment. Thus, Internet marketers have a higher sense of job identity when they have a higher level of psychological resilience and are less likely to experience job burnout. The positive forces stimulated by an individual’s internal resilience can promote work engagement, and the results of the work often make the individual more satisfied, which will ultimately help in achieving subjective career success.

Third, workload has a positive moderating effect on the relationship between psychological resilience and subjective career success. The empirical research results of data analysis show that workload positively moderates the impact of Internet marketers’ psychological resilience on their subjective career success. According to the literature, challenging stressors, such as high workload, can also bring expectations to employees when they consume a large amount of personal resources, such as material and spiritual returns like salary increases, personal growth, and self-realization. Additionally, the empirical data show that the workload perceived by Internet marketers acts as a challenging source of pressure, which is consistent with the core concept of psychological resilience. Hence, when the level of psychological resilience is low, a relatively low workload increases the level of the Internet marketer’s subjective career success perception. When psychological resilience is maintained at a high level, the increase in workload strengthens the transmission mechanism of psychological resilience to work engagement and then to subjective career success, thus enhancing the positive effect.

## Discussion

With the rapid development in digitalization and informatization, the profession of Internet marketers has performed well in absorbing employment, and society should not only focus on the novelty of its job characteristics but also pay attention to their career development process. The difficulties faced by Internet marketers can be used as a direction for future improvement from the perspective of professional psychological quality training and development guarantee, which will ultimately improve the subjective career success cognition level of the Internet marketer group. Thus, based on the research results, it is necessary to provide empirical support for managers to pay attention to fostering Internet marketers’ psychological resilience and, correspondingly, reducing the workload of Internet marketers, and proposing targeted countermeasures to give full play to the positive role of psychological resilience.

First, multiple measures to improve Internet marketers’ psychological resilience. Internet marketers have a high level of psychological resilience to improve subjective career success. Relevant studies have proved that correct self-efficacy and self-acceptance can enhance employees’ psychological resilience and promote their deep development in the professional field. Internet marketers can try to improve their psychological resilience in the following three aspects: first, they should improve their professional identity. Internet marketers, as a new profession, play an important role in the new employment army under the economic environment of live broadcasting. They should highly identify with the mission of engaging in the profession and establish a sense of professional belonging and mission. When Internet marketers are faced with difficulties in their daily work, they should learn to establish the correct style of problem attribution, that is, maintain an optimistic and upward emotional state and seek multichannel and high-efficiency methods to deal with stressful challenges and strengthen emotional regulation ability to improve the level of psychological resilience. Moreover, improve the level of achievement motivation. Renewal of professional skills, emotional experience, and professional knowledge of Internet marketers all promote the level of self-acceptance in the mode of self-renewal and then improve achievement motivation to a certain extent.

Second, focus on multiple dimensions to enhance the work engagement of Internet marketers. The results show that the degree of work engagement has a significant impact on the subjective career success of Internet marketers. Interviews with successful Internet marketers showed that they love their work and are willing to think about how to improve their current work. Interest is the best teacher in the process of work to find and cultivate their own interest in work, stimulate enthusiasm for work, enhance the degree of professional work engagement, and finally succeed in their respective careers. Additionally, the level of work engagement also depends on the Internet marketers’ own problem-solving ability. Groups with high levels of psychological resilience show strong problem-solving ability in the face of difficulties; specifically, they actively think about various solutions to problems, accumulate successful learning experiences from others, and are good at arranging and solving problems based on the difficulty level. Internet marketers can improve their ability to deal with problems through the dual accumulation of theory and practice, such as reading to accumulate more professional knowledge through the practice process to accumulate more experience, thus broadening their horizons and even career and gradually improving their subjective career success cognitive level.

Third, multiple measures to promote rationalization of the workload of Internet marketers. According to this study, when the level of psychological resilience is low, the relatively low workload improves the Internet marketers’ subjective career success cognitive level. By contrast, when there is a high level of psychological resilience, the increase in workload ultimately improves the cognitive level of subjective career success. According to the rule of Yerkes-Dodson, the inverted U between stress and work results means that too high or too low pressure will result in lower work results than expected. For different levels of psychological resilience, a moderate workload can maximize work efficiency. Based on the actual situation, the source of pressure faced by Internet marketers is not only personal but also related to the assessment mechanism of the employment platform and the lack of social security system. Internet marketers can rationally deal with their workload by improving their personal abilities. Because of the novelty of the professional content of Internet marketers, individuals should constantly update their professional skills to cope with the possible working conditions. Hence, Internet marketers can improve their professional abilities, skills, and experience by constantly acquiring new knowledge. Moreover, today’s platform often views the live broadcast time of Internet marketers as one of the work completion standards, although long hours of work bring lower work results. Thus, the employment platform should carefully consider implementing some mandatory evaluation systems and establish a reasonable performance evaluation system for Internet marketers. Additionally, as a new occupation that has just been included in the professional gamut, the workload level of Internet marketers is related to the imperfect security system at the social level. In the future, governments should issue policies related to the professional group to ensure that the group’s basic interests are not hampered at the institutional level.

## Contribution

On the one hand, the theoretical significance of this study entails the extension of the research on psychological resilience to the emerging professional group of Internet marketers to deepen the scope of application and improve the theoretical system of psychological resilience. On the other hand, this study creates a new perspective for the career management research of Internet marketers and attempts to use psychological capital theory and resource preservation theory as the theoretical foundation combined with the work requirement–resource model. It aims to clearly define the psychological resilience of Internet marketers and the subjective career success concept’s connotation of flexible employment under the new employment situation and introduce work engagement and workload variables to explore the mechanism based on the psychological resilience of the Internet marketer group to their subjective career success. Additionally, the psychological resilience of the professional group of Internet marketers has important practical significance and value for individuals or employment platforms. It is reflected as follows: first, when the level of psychological resilience of Internet marketers is high, the degree of investment or concentration at work will be higher than that of Internet marketers with low levels of psychological resilience and individual job satisfaction and other work result variables will also increase accordingly; that is, their own subjective level of career success is also correspondingly higher; second, Internet marketers can exercise individual psychological resilience for some workload undertaken by them so that individuals can demonstrate good work behavior.

## Limitations and future research directions

However, the current study has some limitations. First, the subjects may not be motivated to respond because of the large set of questionnaire items in the survey, which has implications on the number of questionnaires. In future research, the number of samples can be further expanded by adding more distribution channels. Second, the questionnaires in this study are self-assessments by Internet marketers, and individual’s subjective perceptions and the actual situation may be different, which may influence the accuracy of the survey data. Future research can collect data in various ways. For example, the psychological resilience part of Internet marketers can also be scored through social network relationships to collect data. Additionally, this study selects work engagement as the mediating variable and workload as the moderating variable in the model. Although it has enriched the relevant research conclusions, studies on the correlation between the two still need to be expanded (Qi et al., 2016; Wang et al., 2020). Third, the selection of variables and construction of models are based on the current work status of Internet marketers. This research model may be applied to other work characteristics similar to those of Internet marketers. Future studies should explore whether the research model has applicability for occupational groups within a certain range. Fourth, this paper has not delved into the relationship between psychological resilience and subjective career success from the perspective of working years of Internet marketers. Further studies could produce more longitudinal research about investigating the link between psychological resilience and working years. Finally, considering that Internet marketers belong to the typical representatives of flexible employees recognized by China, the research scope can be expanded to foreign flexible employees, not limited to the occupation of Internet marketers. We can also expand the research on the relationship between resilience and subjective career success of foreign flexible employees in the future.

## Data availability statement

The raw data supporting the conclusions of this article will be made available by the authors, without undue reservation.

## Author contributions

TW contributed to the research idea, data analysis, and writing and revising. DG contributed to the data collection, theoretical construction, and writing. Both authors contributed to the article and approved the submitted version.
